# A novel mutation in *ACADVL* causing very long-chain acyl-coenzyme-A dehydrogenase deficiency in a South Asian pediatric patient: a case report and review of the literature

**DOI:** 10.1186/s13256-021-03013-y

**Published:** 2021-09-01

**Authors:** Visvalingam Arunath, Manoj Sanjeewa Liyanarachchi, Sundararajah Gajealan, Eresha Jasinge, Kumudu Weerasekara, Lia Abbasi Moheb

**Affiliations:** 1 Lady Ridgeway Hospital, Colombo 08, Sri Lanka; 2 Centogene, the Rare Disease Company, Am Strande 7, 18055 Rostock, Germany

**Keywords:** Very long-chain acyl-CoA dehydrogenase deficiency, *ACADVL*, Hypoketonemic hypoglycemia, High anion gap metabolic acidosis, Convulsions

## Abstract

**Background:**

Very long-chain acyl-coenzyme-A dehydrogenase deficiency is a rare, severe life-threatening metabolic disorder of mitochondrial fatty acid oxidation, caused by mutations in *ACADVL* gene. Here we present a genetically confirmed case of a South Asian baby girl with severe, early-onset form of very long-chain acyl-coenzyme-A dehydrogenase deficiency due to a novel mutation in *ACADVL* gene.

**Case presentation:**

Index case was the second baby girl of second-degree consanguineous South Asian parents. She had an uncomplicated antenatal period and was born by spontaneous vaginal delivery at term with a birth weight of 2910 g. She had been noted to have fair skin complexion, hypotonia, and 3 cm firm hepatomegaly. Since birth, the baby developed grunting, poor feeding, and recurrent episodes of symptomatic hypoglycemia and convulsions with multiple semiology. Her septic screening and urine ketone bodies were negative. The baby had high anion gap metabolic acidosis and elevated transaminases and serum creatine phosphokinase levels. Echocardiogram at 4 months revealed bilateral ventricular hypertrophy. Acylcarnitine profile revealed elevated concentrations of tetradecanoylcarnitine (C14), tetradecanoylcarnitine C14:1, and C14:1/C16. Unfortunately, the baby died due to intercurrent respiratory illness at 4 months of age. Sequence analysis of *ACADVL* gene in perimortem blood sample revealed homozygous frame shift novel variant NM_001270447.1, c.711_712del p.(Phe237Leufs*38), which confirmed the diagnosis of very long-chain acyl-coenzyme-A dehydrogenase deficiency.

**Conclusions:**

This case demonstrates the importance of early diagnosis and management of very long-chain acyl-coenzyme-A dehydrogenase deficiency in improving the outcome of the patients. Implementation of newborn screening using tandem mass spectrometry in Sri Lanka will be beneficial to reduce the morbidity and mortality of treatable disorders of inborn errors.

## Introduction

Mitochondrial β-oxidation of fatty acids is an essential energy-producing pathway in cardiac and skeletal muscles, mainly during stress conditions. One of the key enzymes involved in this pathway is very long-chain acyl-coenzyme-A dehydrogenase (VLCAD), which is encoded by *ACADVL* gene (OMIM*609575) [1, 2]. Very long-chain acyl-coenzyme-A dehydrogenase deficiency (VLCADD) is caused by pathogenic mutations in *ACADVL* gene, with an incidence range of 1/380,000–1/1,400,000 in Asian region, and hundreds of pathogenic mutations have been reported so far [3–8].

There are very few reported cases of genetically confirmed VLCADD from this region so far. We report a genetically confirmed case of South Asian baby with severe, early-onset form of VLCADD due to a novel mutation in *ACADVL* gene.

## Case presentation

Index case was a baby girl who was the second child of second-degree consanguineous South Asian parents. Her elder sister had died unexpectedly on second day of life, and her postmortem findings were inconclusive.

This baby had an uncomplicated antenatal period and was born by spontaneous vaginal delivery at term with a birth weight of 2910 g. Her initial Apgar scores were 8^1^ 9^5^ 10^10^. Neonatal examination revealed fair skin complexion, hypotonia, and 3 cm firm hepatomegaly. Other system examinations were unremarkable.

At 6 hours of age, the baby developed grunting, lethargy, and poor feeding. Empirical intravenous antibiotics were started, although septic screen was negative.

From her second day of the life, she developed recurrent symptomatic hypoglycemic episodes and convulsions with different semiology. Urine ketone bodies and urine for reducing substances were negative. Venous blood gas analysis revealed pH 7.24 (7.31–7.41), pCO_2_ 34 mmHg (30–40 mmHg), HCO_3_16 mmol/L (22–29 mmol/L), and anion gap 19 mEq/L (8–16 mEq/L) , suggestive of high anion gap metabolic acidosis.

Her general biochemistry investigations showed aspartate transaminase (AST) 465 IU/L (10–34 IU/L), alanine transaminase (ALT) 123 IU/L (13–45 IU/L), and serum creatine phosphokinase (CPK) 3358 IU/L (5–130 IU/L). Other liver function tests, renal function tests, serum uric acid, plasma lactate, and ammonia levels were within normal range.

Chest roentgenogram showed mild cardiomegaly. Electrocardiogram was normal. Echocardiogram at tenth day of life revealed mild septal hypertrophy with small ostium secundum atrial septal defect (ASD). Hepatomegaly was seen on abdominal ultrasound scan. Cerebrospinal fluid analysis including lactate level and ultrasound scan of brain were normal. Electroencephalography revealed slow waves.

Acylcarnitine analysis of dried blood spots, done in a reputed overseas laboratory, revealed elevated concentrations of tetradecanoylcarnitine (C14) of 2.12 μmol/L (0.04–0.5), tetradecanoylcarnitine (C14:1) of 2.61 μmol/L (0.01–0.7), and C14:1/C16 of 0.32 μmol/L (0.01–0.24), suggestive of VLCADD. Plasma amino acid and urine organic acid profiles were normal.

Hypoglycemic episodes had been settled with intravenous fluids and regular breast feeding. Convulsions were controlled with multiple antiepileptic drugs. On day 23 of life, the baby had been discharged from the neonatal intensive care unit with regular oral phenobarbitone doses. The mother had been advised on regular feeding.

Up to 4 months of age, the baby had adequate weight gain without any convulsions or hypoglycemic events. She had been followed up in well-baby clinic and early intervention clinic as she was found to have delayed developmental milestones. Follow-up echocardiogram revealed bilateral ventricular hypertrophy.

At 4 months of age, the baby developed bronchopneumonia that complicated with heart failure secondary to cardiomyopathy, recurrent convulsions, and hypoglycemic events. The baby needed ventilator support and unfortunately died on the sixth day of illness.

Given the strong biochemical results, genetic analysis of the *ACADVL* gene was requested in the perimortem blood sample at CENTOGENE AG. Bidirectional Sanger sequencing of the entire coding region and the highly conserved exon–intron splice junctions was performed. A homozygous frame shift variant NM_001270447.1, c.711_712del p.(Phe237Leufs*38) was detected. The variant is novel and is absent in gnomAD (accession date 14 February 2020). It is classified as likely pathogenic based on the American College of Medical Genetics and Genomic guidelines (ACMG). Parental screening could not be arranged due to financial constraints.

## Discussion

VLCADD is the second most commonly diagnosed disorder of fatty acid oxidation, inherited in an autosomal recessive manner, and associated with a range of phenotypes, varying from asymptomatic patients to severe early-onset multiorgan failure and death. Although various pathogenic mutations in the *ACDAVL* gene have been reported, reports of novel mutations are constantly increasing [3–8]. Genotype–phenotype correlation is not well established in VLCADD; however, there are few reports of potential genotype and phenotype correlation and atypical clinical features with novel mutations [6–11].

Clinical phenotypes of VLCADD vary depending on the age at presentation. Early-onset, cardiac type, which is a severe form, often presents during the neonatal period or early infancy, involves cardiomyopathy and liver dysfunction and has high mortality. Our patient and her elder sibling had the features of severe phenotype, which is associated with null mutations that result in no residual VLCAD activity [9].

Childhood-onset, hepatic type is often associated with intermittent hypoketonemic hypoglycemia with favorable outcome, and late-onset, myopathic type is another milder form that presents during later childhood or adulthood [1]. Although hypoketonemic hypoglycemia and hypotonia are features of childhood- and late-onset phenotypes, our patient had these features, which have been reported in a few severe phenotypes as well [6–8].

Children with VLCADD can present rarely with recurrent convulsions/epileptic encephalopathy, and associated congenital cardiac anomalies are also reported in few cases [6–8, 10, 11]. Interestingly, our child had both these features: recurrent convulsions and ASD with bilateral branch pulmonary artery stenosis.

Reported cases from Europe, America, and Australia have generally mild phenotypes compared with the reported cases from Asian countries, which are more like to the index case and have severe phenotype with high mortality rate. This indicates the ethnic differences in disease presentation. [1, 3–9, 12, 13].

Biochemical examination and acylcarnitine analysis are useful for initial screening tools, usually abnormal during metabolic crisis and normal in levels between the episodes. Hypoketonemic hypoglycemia, high anion gap metabolic acidosis, deranged liver profile, and elevated CPK and plasma ammonia levels are the common biochemical abnormalities [1].

Biochemical examinations, especially CPK and blood gas analysis, are useful screening investigations in low-resource settings. Elevated CPK is usually observed after 1 year of age. Our patient exhibited elevated CPK levels during the neonatal period, which had been reported in few cases with severe phenotypes [6–8].

Acylcarnitine analysis by tandem mass spectrometry (TMS) is included in the routine newborn screening (NBS) programs of most of the developed countries, which reveals elevated C14-acylcarnitine values and ratios. C14:1-acylcarnitine is considered as the primary disease-specific marker of VLCADD [1, 3–8]. Urine organic acid analysis may reveal hypoketonemic dicarboxylic aciduria [13], although normal urine organic acid profile cannot exclude the diagnosis of VLCADD, which had been clearly evident in our baby, who was genetically confirmed VLCADD but repeatedly had completely normal urine organic acid profile.

Thus, each newborn with a suggestive VLCADD by clinically or acylcarnitine profile requires further confirmatory diagnosis by molecular assay by DNA sequencing or direct VLCAD enzyme activity assay in cultured fibroblasts or lymphocytes [1]. As there is a lack of correlation between the biochemical investigations, genetic studies, and presence of clinical symptoms, different confirmatory tests are needed to confirm the diagnosis [3, 4].

Management must be individualized according to each patient’s clinical phenotype and the level of enzyme activity. Recommended treatment options for VLCAD deficiency include a dietary modification and avoiding prolonged fasting. A diet high in carbohydrate and medium-chain triglycerides (MCT) but low in long-chain triglycerides is recommended. l-Carnitine supplementation also has a place in the management; however, its efficacy still remains to be proven [1, 3, 4].

Merinero *et al*. recommended that early initiation of dietary modification in all the cases with abnormal plasma acylcarnitines while pending the results of the enzymatic/molecular analysis can improve the outcomes of the patient [3] and may reverse the cardiac dysfunction in the severe forms of VLCADD [13, 14]. However, Obaid *et al*. reported that even the initiation of dietary modification immediately following the diagnosis with severe phenotypes did not alter the prognosis [7]. As our baby tolerated breastfeeding till the age of 4 months without any metabolic crisis, breastfeeding was continued due to the financial constraints and unavailability of the supplementations.

Interestingly, a few uncommon features of early-onset, severe VLCADD were observed in our case, which could be partly due to novel mutation resulting in hypoketonemic hypoglycemia, hypotonia, recurrent convulsions, congenital heart disease, and elevated CPK levels during the neonatal period. Therefore, the reported novel *ACADVL* mutations will be useful in future clinical work of VLCADD since the diversity of mutations is useful to provide a theoretical basis for clinical diagnosis.

## Conclusion

VLCADD can be a severe life-threatening disorder, if diagnosed late. Therefore, implementation of NBS programs for highly treatable inborn errors of metabolism will allow early diagnosis and prompt intervention that can reduce the infant mortality. Genetic analysis is essential to provide a precise diagnosis, which is crucial for the management of the patient and the family members.

## Data Availability

Not applicable.

## Abbreviations

VLCAD: Very long-chain acyl-coenzyme-A dehydrogenase
VLCADD: Very long-chain acyl-coenzyme-A dehydrogenase deficiency
AST: Aspartate transaminase
ALT: Alanine transaminase
CPK: Creatine phosphokinase
TMS: Tandem mass spectrometry
NBS: Newborn screening
DNA: Deoxyribonucleic acid
MCT: Medium-chain triglycerides

## References

[1] Leslie ND, Valencia CA, Strauss AW, et al. Very long-chain acyl-coenzyme a dehydrogenase deficiency. 2009 May 28 [Updated 2021 May 13]. In: Adam MP, Ardinger HH, Pagon RA, et al. (eds.) *GeneReviews*® [Internet]. Seattle (WA): University of Washington, Seattle; 1993-2019. https://www.ncbi.nlm.nih.gov/books/. 20301763

[2] Shibata N, Hasegawa Y, Yamada K, Kobayashi H, Purevsuren J, Yang Y (2018). Diversity in the incidence and spectrum of organic acidemias, fatty acid oxidation disorders, and amino acid disorders in Asian countries: selective screening vs. expanded newborn screening. Mol Genet Metab Rep.

[3] Merinero B, Alcaide P, Martín-Hernández E, Morais A, García-Silva M, Quijada-Fraile P (2017). Four years’ experience in the diagnosis of very long-chain Acyl-CoA dehydrogenase deficiency in infants detected in three Spanish newborn screening centers. JIMD Rep.

[4] Evans M, Andresen B, Nation J, Boneh A (2016). VLCAD deficiency: follow-up and outcome of patients diagnosed through newborn screening in Victoria. Mol Genet Metab.

[5] Miller MJ, Burrage LC, Gibson JB, Strenk ME, Lose EJ, Bick DP, Elsea SH, Sutton VR, Sun Q, Graham BH, Craigen WJ, Zhang VW, Wong LJ (2015). Recurrent *ACADVL* molecular findings in individuals with a positive newborn screen for very long chain acyl-coA dehydrogenase (VLCAD) deficiency in the United States. Mol Genet Metab.

[6] Rovelli V, Manzoni F, Viau K, Pasquali M, Longo N (2019). Clinical and biochemical outcome of patients with very long-chain acyl-CoA dehydrogenase deficiency. Mol Genet Metab.

[7] Obaid A, Nashabat M, Alfadhel M, Alasmari A, Al Mutairi F, Alswaid A (2017). Clinical, biochemical, and molecular features in 37 Saudi patients with very long chain Acyl CoA dehydrogenase deficiency. JIMD Rep.

[8] Pena LDM, Calcar SCV, Hansen J, Edick MJ, Vockley CW (2016). Outcomes and genotype-phenotype correlations in 52 individuals with VLCAD deficiency diagnosed by NBS and enrolled in the IBEM-IS database. Mol Genet Metab.

[9] Andresen BS, Olpin S, Poorthuis BJ (1999). Clear correlation of genotype with disease phenotype in very-long-chain acyl-CoA dehydrogenase deficiency. Am J Hum Genet.

[10] Joe Y, Phillip L (2013). Metabolic causes of epileptic encephalopathy. Epilepsy Res Treat.

[11] Spiekerkoetter U, Mueller M, Sturm M, Hofmann M, Schneider D (2012). Lethal undiagnosed very long-chain Acyl-CoA dehydrogenase deficiency with mild C14-acylcarnitine abnormalities on newborn screening. JIMD Rep.

[12] Siu WK, Mak CM, Siu SLY, Siu TS, Pang CY (2012). Molecular diagnosis for a fatal case of very long-chain Acyl-CoA dehydrogenase deficiency in Hong Kong Chinese with a novel mutation: a preventable death by newborn screening. Diagn Mol Pathol.

[13] Sharef S, Al-Senaidi K, Joshi S (2013). Successful treatment of cardiomyopathy due to very long-chain Acyl-CoA dehydrogenase deficiency: first case report from Oman with literature review. Oman Med J.

[14] Katz S, Landau Y, Pode-Shakked B, Pessach I, Rubinshtein M, Anikster Y (2017). Cardiac failure in very long chain acyl-CoA dehydrogenase deficiency requiring extracorporeal membrane oxygenation (ECMO) treatment: a case report and review of the literature. Mol Genet Metab Rep.

